# Identifying Disinformation on the Extended Impacts of COVID-19: Methodological Investigation Using a Fuzzy Ranking Ensemble of Natural Language Processing Models

**DOI:** 10.2196/73601

**Published:** 2025-05-21

**Authors:** Jian-An Chen, Wu-Chun Chung, Che-Lun Hung, Chun-Ying Wu

**Affiliations:** 1 Institute of Biomedical Informatics National Yang Ming Chiao Tung University Taipei Taiwan; 2 Department of Information and Computer Engineering Chung Yuan Christian University Taoyuan Taiwan; 3 Health Innovation Center National Yang Ming Chiao Tung University Taipei Taiwan; 4 Microbiota Research Center National Yang Ming Chiao Tung University Taipei Taiwan; 5 College of Medicine China Medical University Taichung Taiwan

**Keywords:** misinformation, COVID-19, ensemble models, fuzzy ranks, language model

## Abstract

**Background:**

During the COVID-19 pandemic, the continuous spread of misinformation on the internet posed an ongoing threat to public trust and understanding of epidemic prevention policies. Although the pandemic is now under control, information regarding the risks of long-term COVID-19 effects and reinfection still needs to be integrated into COVID-19 policies.

**Objective:**

This study aims to develop a robust and generalizable deep learning framework for detecting misinformation related to the prolonged impacts of COVID-19 by integrating pretrained language models (PLMs) with an innovative fuzzy rank-based ensemble approach.

**Methods:**

A comprehensive dataset comprising 566 genuine and 2361 fake samples was curated from reliable open sources and processed using advanced techniques. The dataset was randomly split using the *scikit-learn* package to facilitate both training and evaluation. Deep learning models were trained for 20 epochs on a Tesla T4 for hierarchical attention networks (HANs) and an RTX A5000 (for the other models). To enhance performance, we implemented an ensemble learning strategy that incorporated a reparameterized Gompertz function, which assigned fuzzy ranks based on each model’s prediction confidence for each test case. This method effectively fused outputs from state-of-the-art PLMs such as robustly optimized bidirectional encoder representations from transformers pretraining approach (RoBERTa), decoding-enhanced bidirectional encoder representations from transformers with disentangled attention (DeBERTa), and XLNet.

**Results:**

After training on the dataset, various classification methods were evaluated on the test set, including the fuzzy rank-based method and state-of-the-art large language models. Experimental results reveal that language models, particularly XLNet, outperform traditional approaches that combine term frequency–inverse document frequency features with support vector machine or utilize deep models like HAN. The evaluation metrics—including accuracy, precision, recall, *F*_1_-score, and area under the curve (AUC)—indicated a clear performance advantage for models that had a larger number of parameters. However, this study also highlights that model architecture, training procedures, and optimization techniques are critical determinants of classification effectiveness. XLNet’s permutation language modeling approach enhances bidirectional context understanding, allowing it to surpass even larger models in the bidirectional encoder representations from transformers (BERT) series despite having relatively fewer parameters. Notably, the fuzzy rank-based ensemble method, which combines multiple language models, achieved impressive results on the test set, with an accuracy of 93.52%, a precision of 94.65%, an *F*_1_-score of 96.03%, and an AUC of 97.15%.

**Conclusions:**

The fusion of ensemble learning with PLMs and the Gompertz function, employing fuzzy rank-based methodology, introduces a novel prediction approach with prospects for enhancing accuracy and reliability. Additionally, the experimental results imply that training solely on textual content can yield high prediction accuracy, thereby providing valuable insights into the optimization of fake news detection systems. These findings not only aid in detecting misinformation but also have broader implications for the application of advanced deep learning techniques in public health policy and communication.

## Introduction

### Background

From 2019 to 2022, the global community faced challenges posed by the COVID-19 pandemic. In response, governments worldwide and the World Health Organization (WHO) collaborated extensively to reduce the spread of the virus. An increased demand for trustworthy information sources and accurate health guidance arose during this global health crisis. However, the surge in these informational needs overlapped with the rapid spread of misinformation and false news through social media platforms, leading to widespread public confusion.

The WHO used the term “infodemic” to describe the spread of misinformation during the pandemic [1]. They emphasized the potential threat that such misinformation posed to national epidemic prevention policies. Trust in incorrect or misleading information could result in adverse health behaviors and noncompliance with health policies, worsening the pandemic's challenges.

While the distribution of COVID-19 vaccines contributed to the gradual control of the pandemic, the virus persisted, giving rise to postinfection symptoms known as long COVID, confirmed in at least 10% of people who contracted the virus [2]. Additionally, instances of reinfection after initial recovery were observed, with research from the US Department of Veterans Affairs indicating increased risks of mortality, hospitalization, and postsymptomatic conditions for reinfected patients [3].

Despite the diminishing immediate threat of COVID-19, the ongoing risks associated with long COVID and reinfection make it important to retain public attention on COVID-19–related policies and information. The challenges of fake news and misinformation persist as the world transitions into a postpandemic era coexisting with the virus. Specifically, issues related to long COVID and reinfection continue to be crucial points for misinformation. Therefore, the timely and accurate identification and classification of such misinformation is critical.

### Prior Work

Throughout the COVID-19 pandemic, some studies used machine learning and deep learning techniques to address the challenge of detecting fake news and misinformation.

Patwa et al [4] collected COVID-19–related texts from publicly available fact-checking websites and social media platforms. Their approach involved term frequency–inverse document frequency (TF-IDF) for feature extraction and applying various machine learning algorithms, such as logistic regression, support vector machines, decision trees, and gradient boosting, for the binary classification of fake news. Das et al [5] employed pretrained language models (PLMs), including robustly optimized bidirectional encoder representations from transformers pretraining approach (RoBERTa) and XLNet, for preprocessing and training on the same dataset. By combining predictions from multiple models through voting, they achieved admirable results in the CONSTRAINT 2021 COVID-19 Fake News Detection competition.

Paka et al [6] argued that relying solely on textual features might be insufficient for accurate fake news classification. To address this, they gathered COVID-19–related tweets from Twitter (now known as X), incorporating additional data such as the number of likes for a tweet, URL links, and each poster’s follower count. Introducing a multifeature classification approach for fake news using a cross-stitch unit combined with a long short-term memory architecture enhanced the classification accuracy.

Furthermore, research teams focusing on the Chinese language used deep learning frameworks like recurrent neural network, convolutional neural network, and transformers to classify COVID-19 fake news in Chinese text [7]. These endeavors highlight the global commitment to addressing the infodemic and giving accurate and reliable information.

Additionally, the emergence of large language models (LLMs) based on transformers, such as ChatGPT [8], has become prominent in recent years. These models, capable of understanding natural language and interacting with users, hold the potential to contribute to the development of more robust tools for combating the spread of false news across various domains.

Beyond COVID-19–specific studies, recent research in other domains has demonstrated the effectiveness of machine learning in sentiment analysis and fake news detection. For example, one study proposed a highly efficient technique for polarity classification of X (formerly known as Twitter) data related to COVID-19 fake news [9]. This work applied 5 machine learning classifiers—support vector machine, logistic regression, *k*-nearest neighbor, decision trees, and random forest—to predict whether news was fake or real, thereby completing the natural language processing (NLP) cycle from data corpus to classification. In another study, researchers examined consumer behavior toward online shopping using machine learning [10]. They utilized a count vectorizer to tokenize text documents and build vocabularies, subsequently applying classification models, including *k*-nearest neighbor, random forest, and support vector machine (SVM), to analyze the sentiment scores of the product reviews. A third study focused on opinion mining regarding politics and inflation using a Roman Urdu dataset sourced from Kaggle [11]. The researchers experimented with various text processing techniques and classification algorithms (naive Bayes, Bayes Net, KStar, decision tree, and random forest), demonstrating how attribute selection and preprocessing can improve accuracy. These diverse studies underscore the versatility and potential of machine learning approaches in various contexts, reinforcing and complementing efforts to address the challenges posed by COVID-19 misinformation.

While numerous studies have explored text classification for fake news detection, many have primarily focused on traditional machine learning techniques or earlier deep learning models. However, few studies have compared these approaches with state-of-the-art LLMs that offer advanced natural language understanding capabilities. Moreover, although ensemble methods have been applied in some cases, there remains a lack of investigation into sophisticated ensemble strategies that leverage model confidence rankings and nonlinear fusion techniques to further improve classification performance. This study aims to bridge these gaps by providing a comprehensive comparison between conventional approaches and LLMs and proposing a novel ensemble framework that integrates fuzzy rank-based fusion with the Gompertz function.

### Study Goal

Considering advancements in deep learning technologies and NLP, this study investigates the performance of various deep learning models in detecting fake news. The objective is to provide a scientific and efficient method for fake news detection in the postpandemic era. Texts about long COVID and reinfection were collected from open-source databases and through web crawling, followed by a preprocessing phase to clean and refine the data. Next, various machine learning and deep learning models were trained and evaluated based on their performance after preprocessing. Finally, a fuzzy rank-based ensemble approach combined multiple models. The performance of this ensemble method was then compared with the state-of-the-art LLM methods.

The proposed method achieved an *F*_1_-score of 96.03%, which can significantly help classify misinformation in real time. The results also demonstrate the effectiveness of language models in distinguishing misinformation.

The main contributions of this study are threefold. First, an in-depth analysis of public datasets related to long COVID was conducted, revealing distinct distribution patterns between genuine and fake articles, which provides valuable insights into the nature and propagation of misinformation. Second, a systematic comparison of traditional text classification methods and state-of-the-art PLMs was carried out, delineating the strengths and limitations of each approach in the context of COVID-19 misinformation detection. Third, a novel ensemble method that combines a fuzzy rank approach with the Gompertz function was developed to enhance the classification performance of language models, resulting in a more robust and accurate detection framework.

The rest of this paper is organized as follows. The next section outlines the methodology, including data collection, preprocessing, analysis, machine learning and deep learning models used, proposed ensemble technique, and implementation details. This is followed by the presentation of experimental results and a comparison of different approaches, as well as several real-case inference examples. Finally, the discussion section highlights the principal findings, acknowledges the study’s limitations, and concludes with a summary of key insights.

## Methods

### Overview

The method comprises 4 key stages: data collection, data preprocessing, data analysis, and modeling. Initially, information was collected from diverse publicly available open sources. Given the inherent inconsistency of internet-based open sources, the gathered data underwent a preprocessing phase to enhance their cleanliness. After the preprocessing, a foundational analysis was undertaken to better understand the dataset’s characteristics. Finally, various deep learning models were trained and compared with other text classification methods. We introduced a fuzzy ranking method with the Gompertz function, which adjusts weights based on the confidence scores of each classifier to generate final predictions for each sample. This fusion of ensemble learning and the Gompertz function offers a fresh perspective on prediction methodologies.

### Data Collection

#### Process Explanation

Articles and claims related to COVID-19 were collected from diverse internet sources. The gathered materials underwent a filter phase using keywords associated with long COVID and reinfection, such as chronic, long-term, persistent, after-effects, sequelae, complications, recovery, post covid, post-covid, omicron, subvariant, reinfection, immune, and variant. The resulting dataset, categorized as either “genuine” or “fake,” originated from 3 primary sources: open-source datasets, fact-checking websites, and governmental bodies.

#### Open-Source Dataset

Textbox 1 outlines information related to the open-source datasets.

**Information about the open-source datasets.**

- **Fighting an Infodemic** [4]: This dataset includes COVID-19–related topics from platforms like X (formerly known as Twitter), Facebook, and fact-checking websites. Utilized for the Constraint@AAAI2021 COVID-19 Fake News Detection in English competition, only labeled data from this dataset were used and are available on GitHub [12].
- **CTF (COVID-19 Twitter Fake News)** [6]: Focused on tweets from X, this dataset includes labeled and unlabeled data concerning genuine and fake COVID-19 news. For this study, only the labeled text content data were utilized.
- **CoAID (COVID-19 Health Care Misinformation Data Set)** [13]: This diverse COVID-19 fake news dataset contains news from the internet and social media platforms, user engagements, tweets, and labels appearing on X.
- **FibVID (Fake News Information-Broadcasting Data Set of COVID-19)** [14]: This dataset collects claims from fact-checking websites like Snopes and Politifact, along with related discourse from X. It includes both COVID-19 and non–COVID-19 topics divided into 4 labels. For this study, only data related to COVID-19 from categories 0 and 1 were used.
- **FaCOV (COVID-19 Viral News and Rumors Fact-Check Articles Data Set)** [15]: Collected from 13 English fact-checking websites related to COVID-19, this dataset includes article titles, URLs, claims, and abstracts. Data with 2 category labels were used by merging titles and article contents.

#### Fact-Checking Websites

While open-source databases offer significant support, they often have limitations regarding data timeframes. To overcome these restrictions, web scraping and data cleaning techniques were employed to gather more recent data from verified fact-checking websites such as Snopes [16] and PolitiFact [17], which are certified by the International Fact-Checking Network. The web crawling method was employed to systematically extract articles classified under “CORONAVIRUS” and “COVID-19” from Snopes (data up to August 31, 2023) and PolitiFact (data up to July 31, 2023).

Alongside the article contents, labels were collected for model training. A total of 1500 and 806 texts were extracted from Snopes and PolitiFact, respectively. Subsequently, the collected data underwent keyword filtering to align more closely with the topic.

In PolitiFact, articles are categorized into 6 labels: pants-on-fire, false, barely true, half-true, mostly true, and true. In contrast, Snopes classifies articles into 14 labels: true, mostly true, mixture, mostly false, false, unproven, outdated, miscaptioned, correct-attribution, misattributed, scam, legend, labeled-satire, and lost-legend. Based on the research by Khan et al [18], the labels from diverse sources were reclassified into 2 categories: genuine and fake.

#### Governmental Bodies

Government and public institution websites, such as the WHO and the Centers for Disease Control and Prevention, were also regarded as primary sources. These institutions have consistently distributed up-to-date information and guidelines throughout the pandemic, establishing them as widely acknowledged, reliable, and accurate data sources. Articles related to “long COVID” and “reinfection” were collected from the COVID-19 sections of these websites. Because the original content was often lengthy, ChatGPT [8] was used to refine and reorganize the content into short claims. The structured claims ensured appropriate length and clarity.

Consequently, each claim was labeled as “genuine” owing to its reputable source. The dataset used for model training encapsulated the latest information obtained through these procedural steps. Table 1 presents the filtered sample counts from various data sources.

**Table 1:** Sample size from different sources.

Source	Time until	Sample size, n	Fake label, n	Genuine label, n
CTF^a^	~2021	1292	1130	162
Fighting an Infodemic	~2021	218	62	156
CoAID^b^	~2020	70	0	70
FibVID^c^	~2020	615	318	297
FaCOV^d^	~2021	811	811	0
PolitiFact	~2023	87	42	45
Snopes	~2023	15	9	6
CDC^e^+WHO^f^	~2023	58	0	58
Total	—^g^	3166	2372	794

^a^CTF: COVID-19 Twitter Fake News.

^b^CoAID: COVID-19 Health Care Misinformation Data Set.

^c^FibVID: Fake News Information-Broadcasting Data Set of COVID-19.

^d^FaCOV: COVID-19 Viral News and Rumors Fact-Check Articles Data Set.

^e^CDC: Centers for Disease Control and Prevention.

^f^WHO: World Health Organization.

^g^Not applicable.

### Data Preprocessing

Ensuring the absence of duplicate entries in the merged open-source datasets was crucial. To achieve this, we cross-referenced the data with existing open-source datasets. Any identified duplicate samples were eliminated to reduce redundancy, thereby preventing potential impacts on subsequent model training and analysis performance.

During the preprocessing phase, social media posts and articles, known to include emojis and external links (URLs) frequently, underwent processing to enhance their suitability for distinguishing between genuine and fake news. The *tweet-preprocessor* package was used to eliminate emojis and URLs from the texts because of their low distribution in classification.

All the labels were encoded as “0” (stands for genuine) and “1” (stands for fake), respectively. The distribution of our dataset is illustrated in Figure S1 in Multimedia Appendix 1. As the public data sources exhibited a bias toward the “fake” category, an imbalanced label distribution was observed in the dataset. Therefore, we employed stratified sampling, allocating 10% of the data for testing and 90% for training. This sampling approach ensures consistent label proportions in both the test and training sets, thereby preventing the potential insufficiency of “genuine” samples that may arise from random sampling.

The preprocessing pipeline followed the aforementioned sequence. First, using pandas, duplicate entries were identified and removed, retaining one instance per unique sample. Second, emojis and URLs were removed with *tweet-preprocessor* to enhance textual clarity and clean the text data. Third, we carried out label encoding. Labels were encoded as “0” for genuine and “1” for fake news. Fourth, we split the dataset. Given the imbalanced nature of our data, which had a higher number of “fake” samples, we employed stratified sampling (using *scikit-learn*) to ensure consistent label proportions. Specifically, 90% (2634/2927) of the data were allocated for training, and 10% (293/2927) were allocated for testing.

### Data Analysis

#### Overview

The data analysis included keyword occurrences, sentiment analysis, and subjectivity values. The analysis aimed to get deeper insights from the collected dataset and identified different distributions between genuine and fake articles.

#### Keyword Occurrences

By analyzing the frequency of certain keywords in the data, we were able to gain a better understanding of the public’s interests. In Figure S2 in Multimedia Appendix 1, we can see that over 50% of the fake samples contain the term “immune,” while only a few genuine samples feature it. The term “recovery” is primarily used in genuine samples but is also present in a similar proportion of fake samples. Other keywords such as “variant,” “complication,” and “chronic” also appear more frequently in fake samples. This suggests that such terms are often used to spread fake news.

#### Sentiment Analysis

Sentiment analysis can explain an article’s tone, whether positive, negative, or neutral. Additionally, it can estimate the subjectivity of the text, distinguishing between genuine information and our opinions. To gain deeper insights into the textual data sourced from open-source databases and fact-checking websites, the *TextBlob* package [19] was used for sentiment analysis. *TextBlob*’s sentiment analysis assigns a polarity value ranging from –1 to 1, indicating the sentiment from entirely negative to entirely positive. To classify sentiments based on polarity, we categorized them into the 5 groups, outlined in Textbox 2.

The sentiment analysis revealed the distribution of sentiments within the collected textual data. In Figure S3 in Multimedia Appendix 1, most of the content falls into the “neutral” category. Approximately 65% (1902/2927) of the content in fake texts and 53% (300/566) in genuine texts were classified as “neutral.” Genuine texts showed a higher proportion (204/566, 36%) in the “slightly positive” category compared to fake texts (703/2927, 24%), indicating a tendency for genuine content to include more positive language. Conversely, there were no significant disparities between the distributions of the “strongly negative,” “slightly negative,” and “strongly positive” sentiment categories across the 2 labels.

In addition to sentiment analysis, *TextBlob* also provides a subjectivity value ranging from 0 to 1, indicating the degree of subjectivity within the text, ranging from entirely objective to entirely subjective. To simplify comprehension, we categorized the degree of subjectivity into the following 5 groups, as outlined in Textbox 3.

**Sentiment classification based on polarity score ranges.**

- Strongly negative: polarity values between –1 and –0.5
- Slightly negative: polarity values between –0.5 and –0.1
- Neutral: polarity values between –0.1 and 0.1
- Slightly positive: polarity values between 0.1 and 0.5
- Strongly positive: polarity values between 0.5 and 1

**Subjectivity classification based on score ranges.**

- Low subjectivity: values between 0 and 0.2
- Medium-low subjectivity: values between 0.2 and 0.4
- Medium subjectivity: values between 0.4 and 0.6
- Medium-high subjectivity: values between 0.6 and 0.8
- High subjectivity: values between 0.8 and 1

Based on the analysis illustrated in Figure S4 in Multimedia Appendix 1, the distribution of subjectivity levels between genuine and fake texts appeared similar, mainly concentrated in the “medium subjectivity” category. Genuine texts had 38% (215/566) of their content in this category, while fake texts contained 37% (1083/2927). There was a 5% higher prevalence of fake texts in the “low subjectivity” category than in genuine texts. Conversely, in the “medium-high subjectivity” category, genuine texts surpassed fake texts by a margin of 3%.

While minor differences were observed in the distribution of sentiment and subjectivity between genuine and fake texts, these differences may not serve as definitive classification criteria. Moreover, sentiment analysis encounters challenges in natural language comprehension, such as accurately identifying sarcasm. Therefore, more precise approaches, such as machine learning algorithms and deep learning models, are required to differentiate between genuine and fake news concerning long COVID and reinfections.

### Overview of Models

#### Comparative Evaluation

In this study, a thorough comparison was executed using various classification methods. Traditional machine learning algorithms that used text content features were employed to establish a baseline. Deep learning models, from the hierarchical attention network (HAN) to the bidirectional encoder representations from transformers (BERT) series, were also used to take advantage of the advanced abilities to handle complex textual data information. Additionally, embedding models based on LLMs were used to compare the performance between fee-required models and open-source deep models. The experimental approach helped us evaluate different methods in distinguishing articles with genuine and fake information, especially regarding long-term COVID-19 and reinfections.

#### Support Vector Machine

To establish a baseline model, this study initially selected SVM [20]. In the text classification, linear classifiers are commonly considered strong baseline models. By comparing the performance of the linear classifier with that of deep learning models, we can verify their effectiveness when fine-tuned and employed [21]. To apply SVM for classification tasks, unigram TF-IDF features were generated from the training set data, and the SVM model was trained using these features for binary classification.

#### Hierarchical Attention Network

HAN [22] integrates attention mechanisms at multiple levels, focusing on word and sentence levels, to capture diverse hierarchical structures within documents. We used recurrent neural networks combined with word-attention and sentence-attention layers for text classification, resulting in state-of-the-art performance across 6 datasets. After training this model on the training set, we compared it with the proposed method.

#### Pretrained Language Models

In addition to deep learning models like HAN, this study also fine-tuned PLMs to use their text-understanding capabilities for fake news detection. PLMs, including BERT [23], RoBERTa [24], decoding-enhanced BERT with disentangled attention (DeBERTa) [25], and XLNet [26], are state-of-the-art models widely used in NLP tasks. BERT, introduced by Google, uses masked language modeling and next-sentence prediction to generate contextualized word representations. RoBERTa, an enhancement of BERT by Meta, removes next-sentence prediction and incorporates optimization strategies for improved performance. DeBERTa introduces disentangled attention and enhanced mask decoder mechanisms to further refine self-attention, achieving superior results across NLP tasks. XLNet, developed by Google, employs permutation language modeling and the Transformer-XL architecture to effectively understand bidirectional contextual comprehension, especially in processing long texts, surpassing BERT and RoBERTa in various benchmarks.

#### Large Language Models

The success of LLMs in recent years has significantly advanced applications in the field of NLP. For the classification task in this study, we used OpenAI’s generative pretrained transformer (GPT) embedding model “text-embedding-ada-002” [27,28] and Google’s Gemini embedding model [29] to transform the training set data, combining it with machine learning methods such as SVM for training. Integrating knowledge from large language models and the training dataset helped improve the text classification predictions. Furthermore, we directly applied ChatGPT-4 [30], the state-of-the-art LLM, to infer the texts in the test set. This allowed us to compare the performance of LLMs with our proposed method.

### Fuzzy Rank-Based Ensemble Technique

Ensemble learning combines the strengths of individual models to yield predictions that outperform any contributing model. This study proposed an approach to enhance prediction performance by incorporating the Gompertz function into ensemble learning techniques.

**Figure 1:**
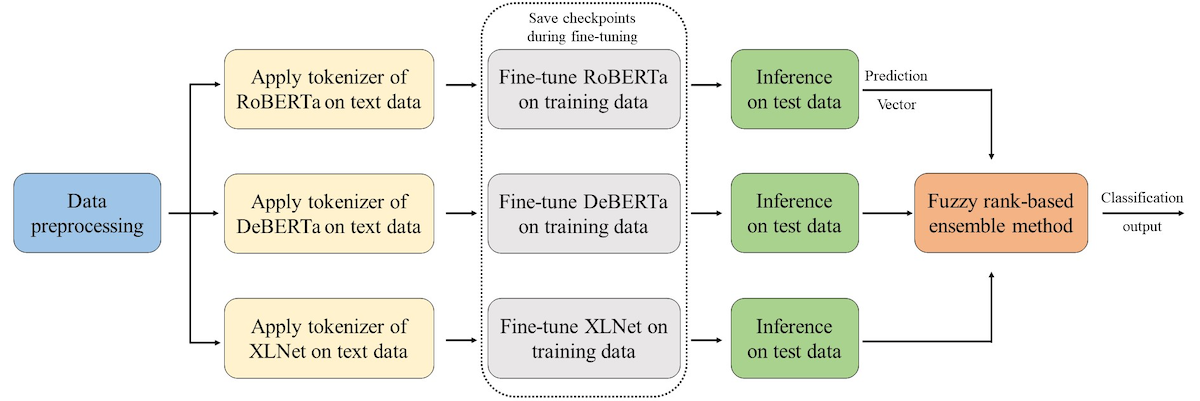
Diagram of the fuzzy ensemble process using multiple language models. DeBERTa: decoding-enhanced bidirectional encoder representations from transformers with disentangled attention; RoBERTa: Robustly optimized bidirectional encoder representations from transformers pretraining approach.

We employed a fuzzy rank-based ensemble technique [31], where the confidence of each classifier in its predictions was given priority for each test case. This differed from traditional ensemble methods like the average or weighted average rules, which assign predefined fixed weights to classifiers. Moreover, the reparameterized Gompertz function was used to compute the fuzzy ranks of each pretrained model for detection. Incorporating state-of-the-art PLMs like RoBERTa, DeBERTa, and XLNet further improved our approach. These PLMs bring advanced language understanding abilities to the ensemble, contributing to its robust performance. Afterward, the predictions of the 3 models were fused. Figure 1 outlines the process of the proposed method.

### Implementation

The data were randomly split using the *scikit-learn* [32] package. This study employed 5-fold cross-validation to train the SVM and determine the best-performing model for the final evaluation. The PLMs were fine-tuned using Hugging Face checkpoints, with the AdamW optimizer [33] and cross-entropy loss. The learning rate was set to 2e-5, and the training was carried out for 20 epochs. HAN was trained for the same number of epochs with its default settings. The training procedure was repeated 5 times with different random initial weights, and checkpoints of models were selected for final evaluation based on the highest validation *F*_1_-score [34]. HAN was trained on the Tesla T4, while all other deep models were trained on the RTX A5000.

### Ethical Considerations

No ethical review was required for this study because it did not involve human participants.

## Results

The model performance was evaluated using well-known metrics: accuracy, precision, recall, *F*_1_-score, and area under the curve (ROC). The results shown in Table 2 compare the proposed fuzzy method with other approaches, including traditional machine learning algorithms, deep learning networks, pretrained networks, and state-of-the-art LLMs.

**Table 2:** Comparison of the performance of different models for test data using various evaluation metrics includes accuracy, precision, recall, F1-score, and AUC.

Model	Accuracy (%)	Precision (%)	Recall (%)	*F*_1_-score (%)	AUC^a^ (%)
TF-IDF^b^ + SVM^c^	89.08	91.46	95.34	93.36	92.02
HAN^d^	90.78	94.09	94.49	94.29	93.09
BERT^e^	91.13	93.75	95.34	94.54	96.31
RoBERTa^f,g^	91.81	93.44	96.61	95.00	96.59
DeBERTa^h,g^	91.81	94.17	95.76	94.96	95.75
XLNet	92.83	94.24	97.03	95.62	96.12
GPT^i^ embedding + SVM	92.83	93.52	97.88	95.65	94.88
Gemini embedding + SVM	91.47	92.71	97.03	94.82	93.27
GPT-4	82.25	91.82	85.59	88.60	N/A^j^
Soft voting	93.17	94.63	97.03	95.82	97.14
Fuzzy rank-based method	93.52	94.65	97.46	96.03	97.15

^a^AUC: area under the curve.

^b^TF-IDF: term frequency–inverse document frequency

^c^SVM: support vector machine.

^d^HAN: hierarchical attention network.

^e^BERT: bidirectional encoder representations from transformers.

^f^RoBERT: robustly optimized BERT pretraining approach.

^g^These models were used in the fuzzy and soft voting methods.

^h^DeBERTa: decoding-enhanced BERT with disentangled attention.

^i^GPT: generative pretrained transformer.

^j^N/A: not applicable.

TF-IDF with SVM achieved an accuracy of 89.08%, a precision of 91.46%, a recall of 95.34%, an *F*_1_-score of 93.36%, and an AUC (area under the curve) of 92.02%. While these metrics indicate acceptable performance, they were still beaten by attention-based deep models like the HAN and BERT series in the experiments.

When comparing HAN with TF-IDF and BERT, we observed that HAN outperformed TF-IDF in terms of accuracy (90.78% vs 89.08%), *F*_1_-score (94.29% vs 93.36%), and AUC (93.09% vs 92.02%), indicating its superior performance in the fake news detection task. However, BERT achieved slightly higher accuracy and AUC than HAN.

**Figure 2:**
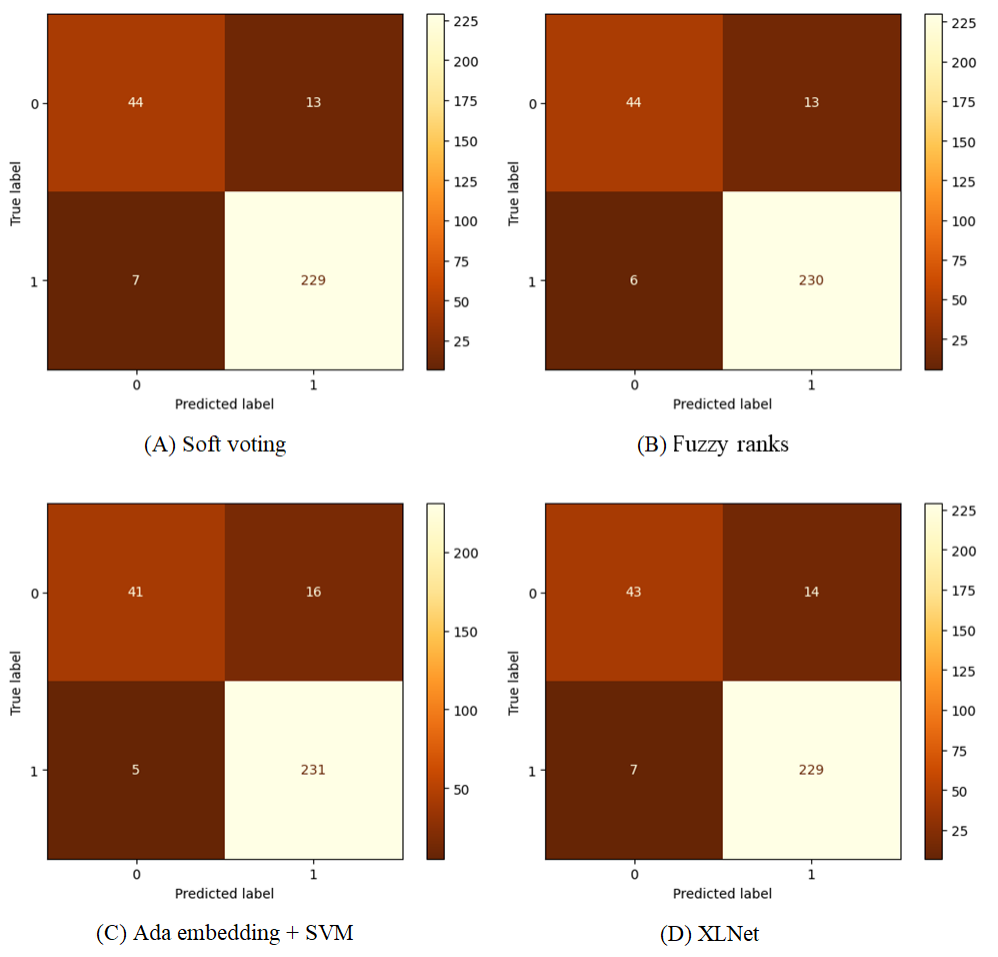
Confusion matrix of (A) soft voting method, (B) fuzzy rank-based method, (C) generative pretrained transformer (GPT) embedding method, and (D) XLNet on the held-out test dataset. SVM: support vector machine.

The fuzzy method achieved impressive results on the test set, with an accuracy of 93.52%, a precision of 94.65%, an *F*_1_-score of 96.03%, and an AUC of 97.15%, representing the highest performance among all the evaluated methods. The conventional soft voting ensemble method on 3 PLMs also yielded a high AUC of 97.14%, while combining the GPT embedding model with SVM yielded the highest recall of 97.88%. This experiment demonstrates the effectiveness of the proposed approach across multiple evaluation metrics, showcasing its potential for robust classification tasks. Figure 2 shows the confusion matrix with the top 4 *F*_1_-scores in the experiment.

Table 3 shows the actual case of using the fuzzy method to detect fake and genuine news that was not included in the test set. We used 4 isolated samples, 2 genuine and 2 fake, varying in length. The result shows that our method accurately detected the genuineness of the content regardless of its length, revealing its robustness across different text lengths.

**Table 3:** Real case inference using fuzzy rank ensemble method.

Content/main claim	Length	Prediction	Ground truth
A German study has revealed that long COVID is linked to the vaccine.	15	Fake	Fake
COVID-19 vaccination before infection is strongly linked to reduced risk of developing long COVID.	15	Genuine	Genuine
Long COVID's causes and risk factors remain a subject of ongoing research, with potential factors including reactivation of SARS-CoV-2 particles, overactive immune responses, and the development of autoantibodies attacking organs. Certain groups, such as those with severe COVID-19 history, underlying health conditions, or lacking vaccination, are at higher risk, alongside other factors like sex, age, initial immune response, and viral variants. Health inequities may also contribute, especially affecting racial or ethnic minority groups and individuals with disabilities.	367	Genuine	Genuine
While Omicron's subvariants find new ways to evade vaccines and destabilize immune systems, another pandemic has overwhelmed officials who are supposed to be in charge of public health. In any case, COVID-19, a novel virus that can wreak havoc with vital organs in the body, continues to evolve at a furious pace. In response, officials have largely abandoned any coherent response, including masking, testing, tracing, and even basic data collection. Yes, the people have been abandoned. So don't expect “normal” to return to your hospital, your airport, your nation, your community, or your life anytime soon.	469	Fake	Fake

## Discussion

### Principal Findings

This study presents a novel approach for detecting misinformation on the long-term effects of COVID-19 by combining state-of-the-art PLMs with a fuzzy rank-based ensemble method that incorporates the Gompertz function. The experimental results demonstrate that using language models, particularly XLNet, for fake news detection outperforms traditional TF-IDF features combined with SVM or deep models like HAN. Some insights can be observed in Table 4, which shows a trend where models with more parameters tend to achieve better classification accuracy. Models such as BERT, RoBERTa, DeBERTa, and XLNet, which possess significantly more parameters than HAN, demonstrated superior performance across various evaluation metrics. Although XLNet had fewer parameters than some models in the BERT series, it still outperformed them in the experiment. This result suggests that classification effectiveness depends not only on the number of parameters but also on the model architecture, training methods, and optimization techniques. XLNet’s success can be attributed to its permutation language modeling approach, which enhances bidirectional context understanding while reducing some limitations in BERT. The fuzzy rank-based ensemble method further enhanced performance by dynamically weighting individual model predictions based on their confidence levels for each test case. This adaptive fusion resulted in an accuracy of 93.52% and an *F*_1_-score of 96.03%.

**Table 4:** Parameter counts for each deep model used in the experiment.

Model	Parameter counts
HAN^a^	2,343,202
BERT^b^	109,483,778
RoBERTa^c^	124,647,170
DeBERTa^d^	139,193,858
XLNet	117,310,466
GPT^e^ embedding	Unknown
Gemini-embedding	Unknown
GPT-4	Unknown

^a^HAN: hierarchical attention network.

^b^BERT: bidirectional encoder representations from transformers.

^c^RoBERTa: robustly optimized BERT pretraining approach.

^d^DeBERTa: decoding-enhanced BERT with disentangled attention.

^e^GPT: generative pretrained transformer.

Additionally, using LLMs’ embedding models led to superior performance than traditional TF-IDF features. In this study, the GPT embedding model performed slightly better than Gemini, possibly due to differences in the length of the embedding vectors. GPT defaulted to 1536, while Gemini defaulted to 768. Despite yielding acceptable outcomes, directly using GPT-4 fell short compared to training SVM on vector-transformed training data using embedding models. This result implies that LLMs still benefit from training data in fake news detection tasks. Although the LLMs’ embedding models showed remarkable performance in the experiment, accessing this kind of embedding model via an application programming interface incurs charges. However, the fuzzy method can be combined with open-source PLMs to achieve even better results. Moreover, compared to using a single language model or soft voting method with predefined weights, the fuzzy fusion-based technique allowed us to determine ensemble model weights for each test case, resulting in superior performance.

### Limitations

One limitation of the study is the presence of data imbalance, which suggests a potential bias toward the prevalence of fake information on the internet. Addressing this issue would require gathering a more extensive and up-to-date dataset of genuine information to achieve a better balance and representativeness in the training data.

### Conclusions

This study provides a comprehensive investigation into the detection of COVID-19–related misinformation by leveraging advanced deep learning techniques. An in-depth analysis of open-source datasets related to long COVID revealed distinct distribution patterns between genuine and fake articles, offering valuable insights into the nature and propagation of misinformation. A systematic comparison between traditional text classification methods and state-of-the-art PLMs highlighted the strengths and limitations of each approach when applied to misinformation detection. Furthermore, the development of a novel ensemble method that integrates a fuzzy rank approach with the Gompertz function significantly enhanced the accuracy and robustness of the text classification.

By developing an ensemble method that integrates fuzzy ranking with the Gompertz function, this research introduces a novel perspective on enhancing classification stability and accuracy. The proposed approach moves beyond simple majority voting or static weight assignments by incorporating dynamic confidence-based fusion, offering a refined framework for complex classification tasks in NLP.

Beyond its methodological contributions, this study holds practical relevance for addressing real-world challenges. The proposed detection system, which performs well using text-only inputs, demonstrates strong potential as a scalable tool for the real-time monitoring of internet misinformation. By helping to distinguish between credible and misleading information, the system may support public health efforts, reduce confusion among the public, and contribute to more transparent digital communication in health-related discourse.

## Appendix

Multimedia Appendix 1 Additional figures.

## Abbreviations

AUC: area under the curve
BERT: bidirectional encoder representations from transformers
DeBERTa: decoding-enhanced BERT with disentangled attention
GPT: generative pretrained transformer
HAN: hierarchical attention network
LLM: large language model
NLP: natural language processing
PLM: pretrained language model
RoBERTa: robustly optimized BERT pretraining approach
SVM: support vector machine
TF-IDF: term frequency–inverse document frequency
WHO: World Health Organization

## Data Availability

The code and datasets generated or analyzed during this study are not publicly available due to an ongoing patent application but may be available from the corresponding author on reasonable request.
