# Supplementary material for: Identifying Disinformation on the Extended Impacts of COVID-19: Methodological Investigation Using a Fuzzy Ranking Ensemble of Natural Language Processing Models
Source: J Med Internet Res. 2025 May 21;27:e73601. doi: 10.2196/73601 (PMC12138316; doi:10.2196/73601)

73601 Multimedia Appendices

Figure S1. Data distribution after preprocessing. 0 stands for genuine, and 1 stands for fake.
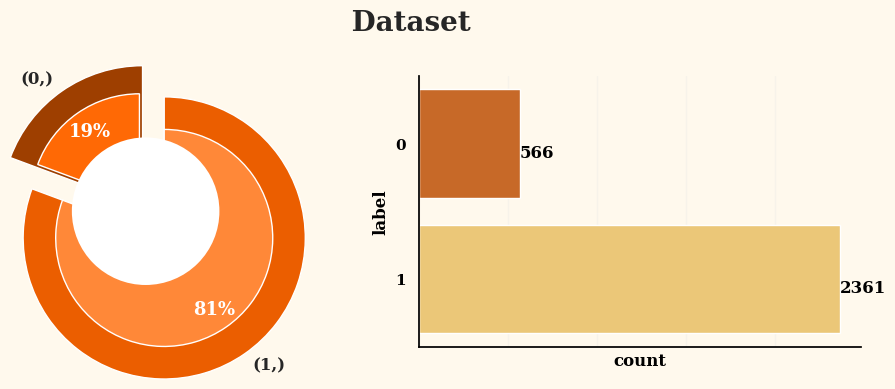


Figure S2. Number of keyword occurrences by label.


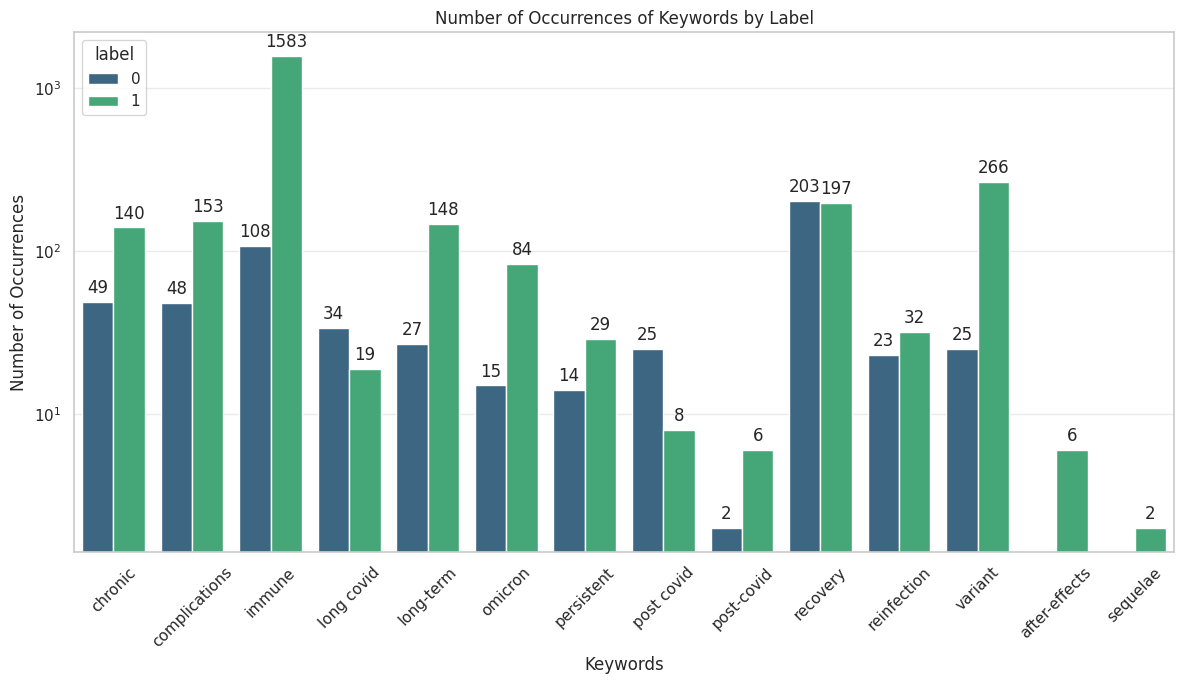


Figure S3. Data distribution (percentage) of different sentiment polarity groups.


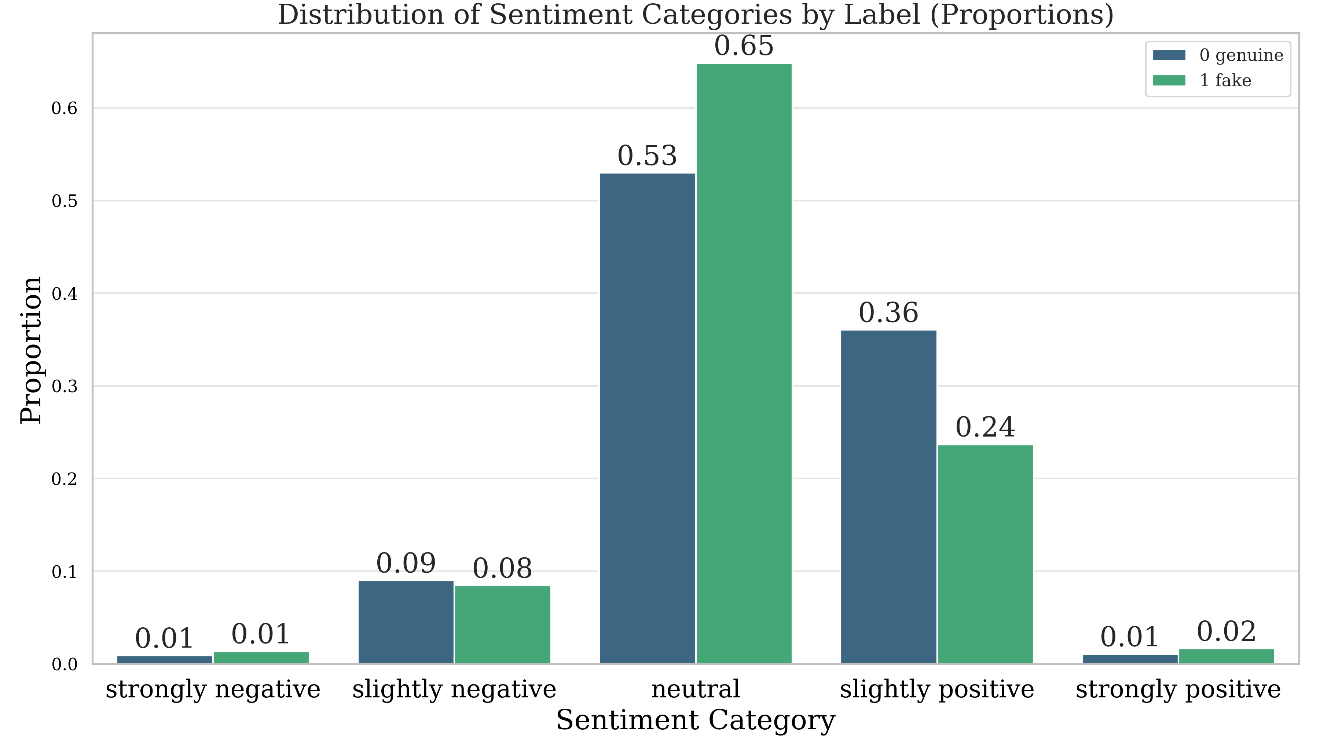


Figure S4. Data distribution (percentage) of different subjectivity groups.


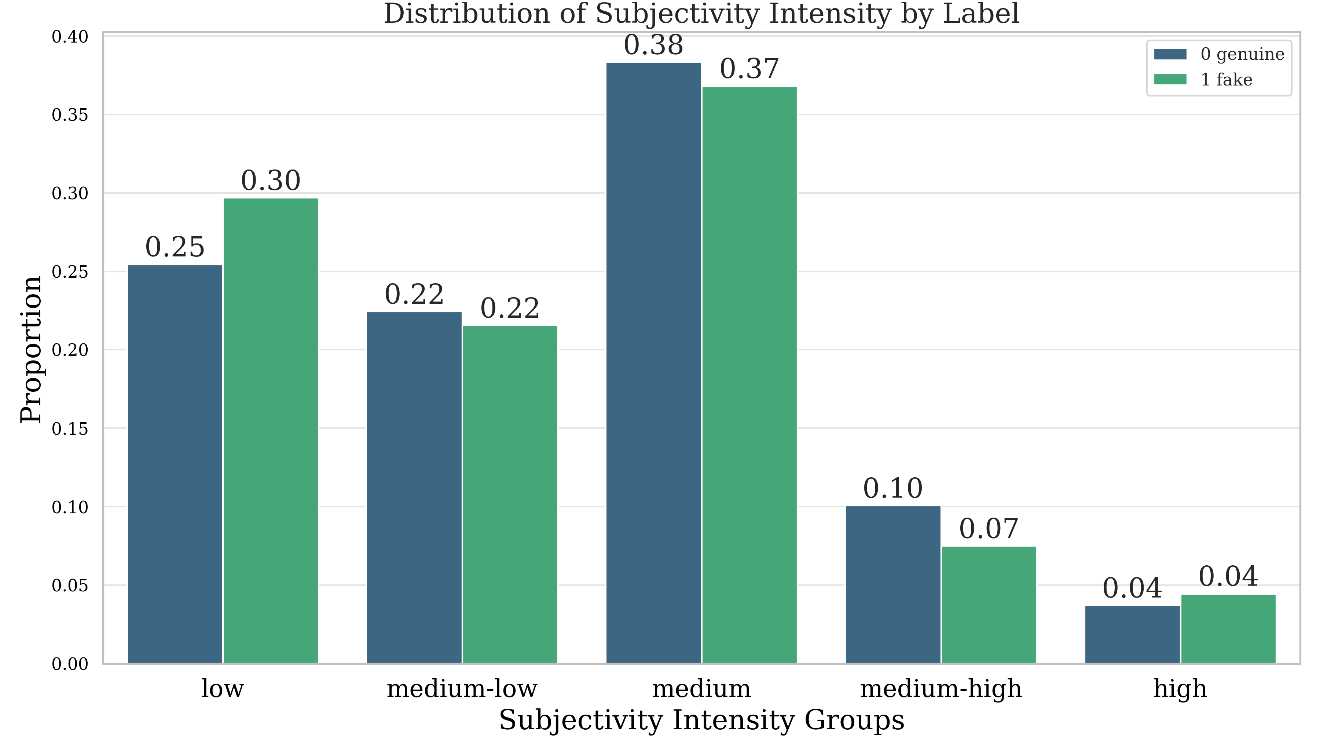

Supplement: Multimedia Appendix 1 [file jmir_v27i1e73601_app1.docx]
